# Biogenic Selenium Nanoparticles and Their Anticancer Effects Pertaining to Probiotic Bacteria—A Review

**DOI:** 10.3390/antiox11101916

**Published:** 2022-09-27

**Authors:** Asad Ullah, Jing Mu, Fenghuan Wang, Malik Wajid Hussain Chan, Xian Yin, Yonghong Liao, Zulfiqar Ali Mirani, Syed Sebt-e-Hassan, Sadar Aslam, Muhammad Naveed, Muhammad Naseem Khan, Zakia Khatoon, Mohib Reza Kazmi

**Affiliations:** 1 Beijing Advanced Innovation Center for Food Nutrition and Human Health, Beijing Technology & Business University (BTBU), Beijing 100048, China; 2 School of Light Industry, Beijing Technology & Business University (BTBU), Beijing 100048, China; 3 Food and Marine Resources Research Center, Pakistan Council of Scientific and Industrial Research Laboratories Complex, Karachi 75280, Pakistan; 4 Department of Chemistry, Faculty of Science, Federal Urdu University of Arts, Science and Technology, Campus Gulshan-e-Iqbal, Karachi 75300, Pakistan; 5 Department of Chemistry, Karakoram International University, Gilgit 15100, Gilgit-Baltistan, Pakistan; 6 Institute of Marine Science, University of Karachi, Karachi 75270, Pakistan; 7 Department of Microbiology, University of Karachi, Karachi 75270, Pakistan; 8 Department of Applied Chemistry, Faculty of Science, University of Karachi, Karachi 75270, Pakistan

**Keywords:** selenium nanoparticles, anticancer, probiotic bacteria, breast, lungs, prostate cancer

## Abstract

Selenium nanoparticles (SeNPs) can be produced by biogenic, physical, and chemical processes. The physical and chemical processes have hazardous effects. However, biogenic synthesis (by microorganisms) is an eco-friendly and economical technique that is non-toxic to human and animal health. The mechanism for biogenic SeNPs from microorganisms is still not well understood. Over the past two decades, extensive research has been conducted on the nutritional and therapeutic applications of biogenic SeNPs. The research revealed that biogenic SeNPs are considered novel competitors in the pharmaceutical and food industries, as they have been shown to be virtually non-toxic when used in medical practice and as dietary supplements and release only trace amounts of Se ions when ingested. Various pathogenic and probiotic/nonpathogenic bacteria are used for the biogenic synthesis of SeNPs. However, in the case of biosynthesis by pathogenic bacteria, extraction and purification techniques are required for further useful applications of these biogenic SeNPs. This review focuses on the applications of SeNPs (derived from probiotic/nonpathogenic organisms) as promising anticancer agents. This review describes that SeNPs derived from probiotic/nonpathogenic organisms are considered safe for human consumption. These biogenic SeNPs reduce oxidative stress in the human body and have also been shown to be effective against breast, prostate, lung, liver, and colon cancers. This review provides helpful information on the safe use of biogenic SeNPs and their economic importance for dietary and therapeutic purposes, especially as anticancer agents.

## 1. Introduction

Selenium (Se) is a natural, non-metallic, essential micronutrient for humans that is mainly consumed through diet and/or supplements [1]. In nature, Se occurs in four oxidation states: Se^6+^ (selenate), Se^4+^ (selenite), Se^2−^ (selenide), and Se^0^ (elemental Se). The biological and toxicological effects of Se, whether in anthropogenic or natural environments, depend on a specific chemical state [2]. The zero oxidation state of Se (Se^0^) found in selenium nanoparticles (SeNPs) shows lower toxicity and admirable bioavailability compared to other oxidation states of Se^6+^ Se^4+^ and Se^2−^ [3,4]. However, biogenic SeNPs have been shown to be safe. So the biogenic SeNPs are gaining interest and recent experiments have shown that they are better than synthetic SeNPs and even the other organic and inorganic Se species used in the past [5,6,7].

In this decade, biogenic SeNPs are gaining importance in medicine due to their high therapeutic value. SeNPs are synthesized by physical, chemical, and biogenic methods. Compared to other approaches (physical and chemical), SeNPs prepared by biogenic methods are more stable and do not aggregate due to the coating of biomolecules. Consequently, no additional stabilizing agents are required [8,9,10,11]. Non-biogenic processes require high temperatures, low pH, and undesirable chemicals [10] that could make the nanoparticles toxic, making them risky for human consumption. Biogenic SeNPs contain non-toxic material and are safe for human use. They are also eco-friendly and have no toxic effects on the natural ecosystem [8,12,13,14]. The microbes involved in biogenic synthesis are bacteria, fungi, algae, actinobacteria, and yeasts. Various bacterial species have been used extensively for the biosynthesis of SeNPs. Pathogenic species synthesizing SeNPs include *Escherichia coli* ATCC 35218 [15], recombinant *E. coli* [16], *Ralstonia eutropha* [17], *Enterobacter cloacae* Z0206 [18], *Pseudomonas aeruginosa* ATCC 27853 [19], *Klebsiella pneumonia* [20,21], *Pantoea agglomerans* [4], *Zooglea ramigera* [22], *Rhodopseudomonas palustris* strain N [23], *Shewanella* sp. HN-41 [24], *Azoarcus* sp. CIB [25], *Burkholderia fungorum* [26], *Stenotrophomonas maltophilia* [27], *Staphylococcus carnosus* [28] etc. Probiotic/nonpathogenic bacteria capable of synthesizing SeNPs comprise *Lactobacillus casei* [21,29,30], *Lactobacillus acidophilus* LA-5, *Lactobacillus helveticus* LH-B02, *Streptococcus thermophilus*, *Bifidobacterium bifidum* BB-12 [29], *Enterococcus faecalis* [31], *Bacillus* sp. MSh-1 [32,33], *Bacillus subtilis* [34], *Bacillus mycoides* SelTE01 [35], *Bacillus licheniformis* JS2 [36], *Bacillus megaterium* [37], *Streptomyces* sp. ES2-5 [38] etc.

**Figure 1:**
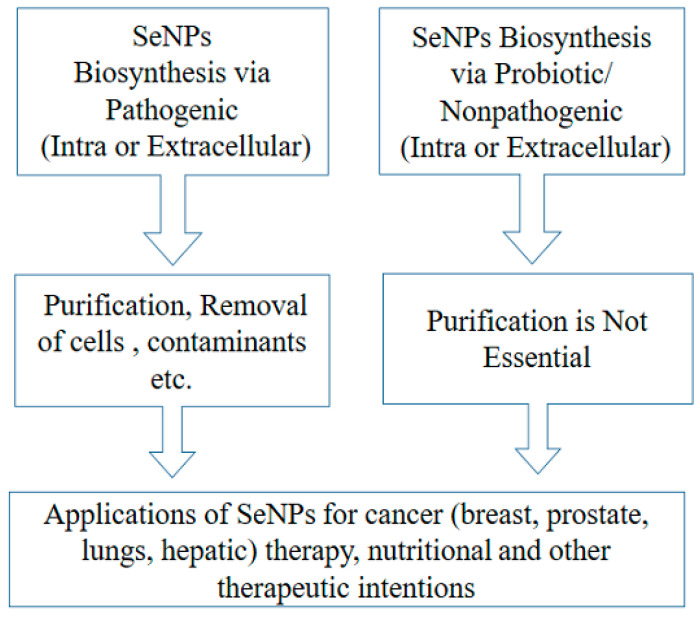
Process handling of biogenic SeNPs and their application as anticancer agents.

The nanomaterials synthesized by microbes are versatile and have advantages over conventional methods [39]. Selenium-resistant bacteria convert Se intracellularly to a non-toxic organic form until their Se tolerance limit is exceeded, and they begin to produce the nano-sized Se particles (Se^0^) (intracellularly or extracellularly). For example, in our recent studies, a probiotic *Bacillus subtilis* (BSN313) accumulated Se in its cell, up to 12 µg/mL, and beyond this level, it began to produce SeNPs [40,41]. It is known that SeNPs are only synthesized by those bacteria that have the ability to resist selenium metal. The synthesis of these nanoparticles can occur extracellularly, intracellularly, or from the membrane-bound cell organelles. For example, *Escherichia coli* can convert the metallic form of selenium into a useful biogenic form by the cytoplasmic membrane-bound organelles and deposit it on the outer wall of *E. coli*. It has been suggested that in addition to *E. coli*, many other bacterial species (both Gram-negative and Gram-positive) are also capable of synthesizing SeNPs, e.g., *Veillonella atypica*, *Pseudomonas* sp, and *B. subtilis* strains, etc. [8]. The anticancer activity of SeNPs has been extensively revealed through various in vitro [42,43,44,45,46,47,48,49,50,51] and in vivo studies [52,53,54,55,56,57,58,59]. All biogenic SeNPs have good therapeutic value, including anticancer effects. However, SeNPs produced by pathogenic bacteria need to be purified before use. (Figure 1). Therefore, the use of common probiotic bacteria in the biosynthesis of SeNPs has been recommended as a useful tool for cancer prevention and treatment. Their use has been shown to be safe and cost-effective, as conventional chemical treatment methods increase the cost of the drug (SeNPs) and have harmful effects on human health and the environment, and they are recommended as expected supportive immunotherapeutic agents in cancer treatment [60,61].

The current study further summarized the information on the anticancer effects of SeNPs produced by probiotic/nonpathogenic bacteria. The, SeNPs produced from probiotic/non-pathogenicbacteria, together with their live or dried biomass, could enhance the anticarcinogenic potential. However, there is no review article in the literature that provided such information in detail. Therefore, this review focuses on the anticancer effects of SeNPs produced from probiotic/nonpathogenic bacterial sources.

## 2. Selenium Requirement and SeNPs

The recommended daily allowance (RDA) for Se for healthy adult women and men is set by the United States as 55 µg/day, and this value is associated with the highest level of antioxidant enzyme activity [62,63]. A slightly higher and more precise RDA of 70 µg/day for men, 60 µg/day for women, and 75 µg/day for lactating women has been proposed by the European Food Safety Authority [64]. In contrast, the World Health Organization (WHO) and the Food and Agriculture Organization (FAO) have set the upper tolerable intake levels (UL) at 400 µg/day for individuals over 14 years of age [39].

Selenium is an essential trace element required for good health and physical development. Deficiency of Se can cause muscular dystrophy, hypothyroidism, cardiomyopathy, suppression of immunity, Keshan and Kashin-Beck disease. Due to fetal growth and development, selenium supplementation is required during pregnancy and lactation, and deficiency can lead to fetal gene mutation or neonatal hemolytic anemia [65]. When taken in excess, it causes toxicity that can lead to severe damage. Thus, excessive intake of >200% of RDA leads to the occurrence of type 2 diabetes, melanoma, cancers of the oropharynx, urinary tract, and lymphoid [66]. When the selenium level in the human body is too high, endothelial function will be impaired by activating endoplasmic reticulum stress response and increasing the production of reactive oxygen species (ROS). However, endothelial dysfunction is the first step toward atherosclerosis [67]. If it has received a high selenium intake from birth or at a very young age, it may change the composition of gut microflora and excess selenium is excreted, which in turn reduces selenium toxicity [68,69]. Se deficiency is common in most regions of the world, so Se supplementation is recommended for individuals from countries and regions with selenium deficiency [70]. Se deficiency can be reduced or prevented by supplementation with inorganic or organic Se sources. However, SeNPs are believed to be novel and reliable candidates as alternatives to other Se species in dietary supplements and medical practice, due to their lower toxicity and ability to release Se when used [66,71].

## 3. Hypothesized Mechanism of Biogenic Formation of SeNPs

The mechanism of bacterial SeNPs biosynthesis is not well understood to date, but some studies have provided ample information to understand this process and have proposed a 7-step SeNPs biosynthesis in *Bacillus mycoides* SeITE01 [72]. (1). Precipitation of selenite (SeO_3_^2−^) in the cytosol as SeNPs, either by the possible activity of low molecular weight thiols containing bacillithiol (BSH) or by the Trx/TrxRed scheme. (2). Intracellular selenite reduction and formation of SeNPs as a result of membrane-bound reductase activity. (3). Discharge of intracellularly produced SeNPs via subsequent cell lysis. (4). Membrane-bound reductases may also catalyze extracellular selenite precipitation. (5). Compounds having thiol groups and peptides are possibly released from the cell and react with selenite. (6). In the presence of NADH extracellular proteins are also capable to mediate selenite precipitation. (7). Nascent SeNPs are inherently unstable due to their larger surface area, so they can continue to mature via the Ostwald growth mechanism to reach their lower-energy state.

BSH-producing bacteria may have analogous enzymes such as Bacillus redoxin (Brx) instead of glutaredoxin (Grx). However, the reductase system suitable for BSH formation in Bacillus species is not yet fully known. Nevertheless, the appropriate involvement of Brx-like proteins in Gram-negative bacteria could have a corresponding detoxification pathway of reduction of SeO_3_^2−^ (selenite) to Se^0^ followed by cellular compression in the form of selenium nanoparticles [73,74].

Debieux et al. [75] also proposed a two-step bacterial (Thauera selenatis) progression mechanism for SeNPs assembly involving glutathione.

1. SeO_4_^2−^ + 2e^–^ + 2H^+^ ⇆ SeO_3_^2−^ + H_2_O
2. SeO_3_^2−^ + 4e^–^ + 6H^+^ ⇆ Se^0^ + 3H_2_O

Currently, they have further demonstrated that biogenic SeNPs were stabilized in the presence of Sef A protein (almost 95 kDa), which may play a potential role in the production of SeNPs.

Various bacterial mechanisms for the reduction of selenates and selenites may involve one or more metabolic pathways involving enzymes and/or proteins. In several microbes, nitrite and nitrate reductases are responsible for denitrification and are similarly involved in the reduction of Se ^+IV^ compounds [76]. The reduction of selenite to zero-valent Se could possibly also occur by catalysis of nitrate reductase from *E. coli* [77] or else nitrite reductase (from *Rhizobium*) [78]. Therefore, the ability to reduce selenates, selenites and tellurites is mainly associated with denitrification [77]. A selenate reductase originated from *T. selenatis* was found involved in selenate reduction [79].

Bacterial Se reduction has been largely elucidated, but the mechanism for SeNPs assembly, morphology, and stabilization is not yet clear. It is therefore recommended that organized studies be conducted in this regard.

## 4. SeNPs as Potential Candidate for Dietary Supplement

The metabolism and absorption of nanoparticles in the gastrointestinal tract (GIT) have been well described in previous literature [80,81,82]. The thickness of the mucus layer varies between 480 and 800 µm in the colon and 200 µm in the small intestine [83]. The pore size in the mucus layer is about 100 nm, which may allow the passage of nanoparticles through the layer [84].

After infiltration of nanoparticles through the mucus layer, they inevitably encounter the thickness of epithelial cells of 20–800 µm, depending on their position in the GIT. The epithelial cells are polarized and divided into numerous layers, which are usually connected by junctions and are essential for paracellular transport and tissue elasticity [75]. A likely pathway for nanoparticle entry into cells could occur under the influence of active or passive transport. The major pathways for nanoparticles, such as endocytosis, macropinocytosis, clathrin- and caveolae-mediated, and non-clathrin- and non-caveolae-mediated. Nanoparticles can also be ejected from cell tissues by transcytosis or exocytosis [84,85,86].

The intake, absorption, transport, and excretion of SeNPs from the human body can be influenced by several factors, including surface properties and size. In addition, interaction with proteins depends on the external chemistry of the nanoparticles. Since proteins are polar compounds, interaction with nanoparticles results in either absorption/accumulation (attachment to the surface of the protein) or excretion (by repulsion), depending on the nature of the charge. Fluctuations in gastrointestinal pH can cause the accumulation of SeNPs in GIT [87]. To increase the stability and transport capacity of SeNPs, polymers such as chitosan could be used [88]. Chitosan is a positively charged common polysaccharide that is widely used as a carrier for many drugs due to its low toxicity and ability to increase drug bioavailability and bio-adhesion [89]. The resistance to digestive enzymes and the associated bioactivity have been studied for the drugs modified with chitosan Chitosan-modified SeNPs showed excellent stability in terms of pH and enzyme activity under simulated GIT conditions [90].

Advanced research suggests that the size of SeNPs plays the main role in their biological activities. Usually, particles of smaller size are more effective than larger ones [91,92]. Smeller SeNPs increase their biological activity by enhancing the action of thioredoxin reductase and selenoenzymes peroxidase [93,94]. Furthermore, smaller SeNPs have much lower toxicity than larger ones [95].

Numerous studies have also addressed the effect of SeNPs on various animals. Subsequent exposure to oxidative stress or uncertainty due to toxic atmospheres indicates encouraging results for the use of SeNPs as a novel dietary supplement [96,97,98,99,100,101]. Instead, it is suggested that further in-depth research on effective dose supplements of SeNPs should be conducted.

## 5. SeNPs as Anticancer Agents beyond the Toxicity

It is well known that long-term intake of selenium in excess amounts can have adverse physiological effects on humans. Long-term intake of selenium in excess amounts leads to rapid development of severe gastrointestinal and neurological symptoms, followed by acute respiratory failure, myocardial infarction, and renal failure, and may increase the risk of cancer [96,97,98,102,103,104]. The Food and Nutrition Board set a tolerable upper Se intake level (UL) for adults at 400 μg/day [105,106]. Toxicity testing is an important concern in the improvement in selenium-containing anticancer drugs. Various forms of selenium could serve as pro-oxidant toxic agents and promote DNA strand disruption and necrosis of cancer cells [46]. Among the many types of nanoparticles, SeNPs have selective anticancer activity on cancer cells and low toxicity to normal cells [107,108,109,110,111,112,113,114,115,116,117]. They exhibit low toxicity, better bioavailability and higher activity as compared to organic and inorganic selenium compounds [109]. In the peritoneal cavity, the clearance rate of SeNPs was slower than in plasma, so they may activate an enhanced drug concentration near the cancer-related peritoneal cavity. Therefore, by maintaining a low total drug level, the use of active doses in the cancer model was expected to be less cause of suspected adverse reactions [110].

One of the most common symptoms of chronic Se toxicity is low food intake and appetite, resulting in significant weight loss [111]. SeNPs at a dose of 0.7 mg Se/kg inhibited cell proliferation by 99% in the case of smaller size nanospheres, with no toxicity observed [110]. A dose of 4 mg/kg SeNPs did not promote loss of body weight and pathological changes in the liver of animals [112]. Similarly, Zhang et al. [113] and Srivastava and Kowshik [114] have reported that SeNPs have potential chemopreventive activity with remarkably low toxicity risk. Among most nanomaterials, SeNPs are considered to be the most promising nanoparticles due to their greater biocompatibility and higher anticarcinogenic activity [115,116].

### 5.1. Presumed Anticancer Mechanism

**Figure 2:**
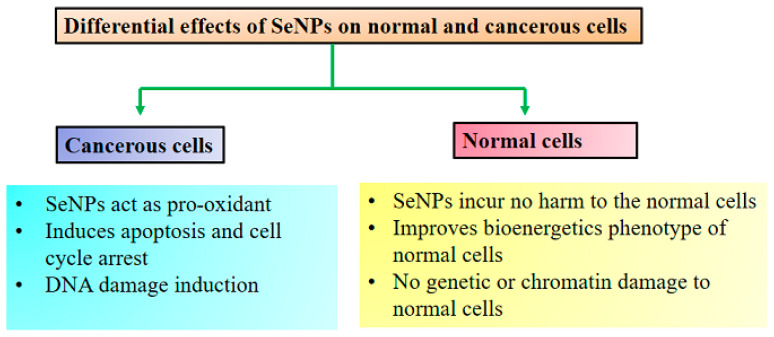
Differential anticancer effect of SeNPs. Adopted from Khurana et al. [106].

**Figure 3:**
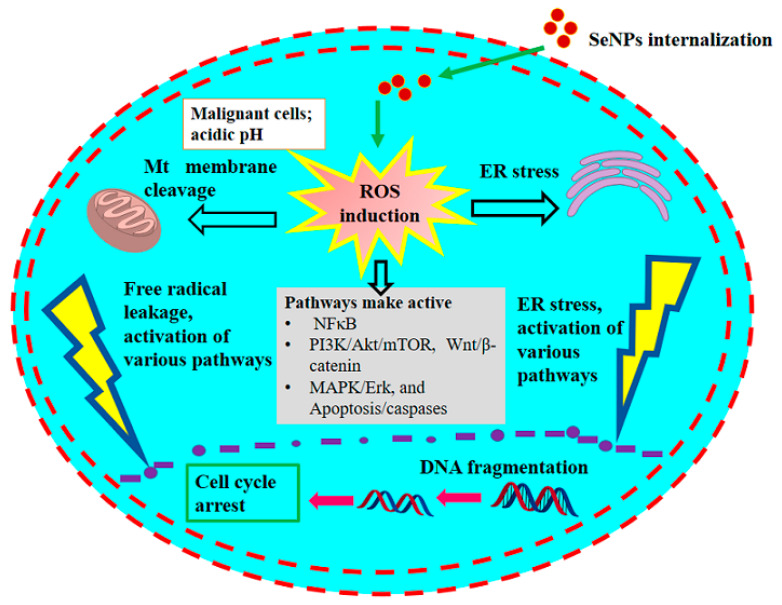
The anticancer mechanism of SeNPs in cancerous cells. Updated as from Khurana et al. [106].

The anticancer effects of SeNPs remain unclear to date; however, the presumed differential effects and cellular anticancer mechanism of SeNPs are outlined (Figure 1, Figure 2 and Figure 3). In general, biogenic SeNPs have shown more differential cytotoxicity on cancer cells compared with normal cells [107,117]. The chemopreventive influence of SeNPs and their presumed anticancer mechanisms were recently well evaluated by Menon et al. [108] and Khurana et al. [107]. Cancer cells have an acidic pH with redox perturbations, and this internal environment leads to a preoxidative transformation of SeNPs, triggering an increased production of free radicals. This leads on the one hand to a disruption of the mitochondrial membrane, causing mitochondrial (Mt) proteins to leak out and on the other hand to a stress of the endoplasmic reticulum (ER). Disruption of the Mt membrane results in the efflux of various proteins and triggers apoptosis through the activation of caspases (a family of protease enzymes that play an essential role in programmed cell death). This stress state of the cell coordinates the activation of several molecular signaling pathways, including MAPK/Erk, PI3K/Akt/mTOR, NFκB, Wnt/β-catenin, and apoptosis pathways. The NFκB pathway triggers oxidative stress and disrupts cellular homeostasis through inflammation. The PI3K/Akt/mTOR, MAPK/Erk, VEGF, and Wnt/β-catenin signaling pathways are important for oncogenic signaling, and their modulation by SeNPs impairs cellular proliferation and hinders growth-promoting signaling in the cancer cell microenvironment. Moreover, SeNPs reduced angiogenic signaling in tumor cells, further inhibiting proliferation and growth. The consolidation of these disruptive cellular events begins with the destruction of DNA, leading to cell cycle arrest and eventually cell death.

The next part of this review deals with the anticancer effects of SeNPs produced using probiotic/nonpathogenic bacteria and has been presented in Table 1, while the details are discussed in detail in the following sections.

### 5.2. Effective against Breast Cancer

Breast carcinoma is the most common malignancy in women throughout the world, with significant regional and racial disparities [128,129,130]. The incidence of this disease in Pakistan is 2.5 times higher than in India and Iran. Approximately 1 million cases of breast cancer in women are reported annually, mainly in industrialized countries [131]. In the past decades, it has become clear that matrix metalloproteinases (MMPs) play an important role in tumor development, metastasis, and incidence. MMPs can serve as good biomarkers for breast carcinoma, and their levels are related to the stage of the disease [132,133]. Proteolysis of the basement membrane and extracellular matrix, particularly collagen IV, is one of the essential processes involved in breast cancer metastasis [134], and this proteolytic degradation occurs through the action of various MMPs. In addition to metastasis, MMPs are also involved in tumor growth, angiogenesis, and vasculogenesis [132].

*Bacillus* sp. MSh-1-produced SeNPs with a particle size of 80–220 nm showed cytotoxic and inhibitory effects on human MMP-2 expression in HT-1080 (fibrosarcoma cell line) in a dose-dependent manner [33]. This inhibitory effect of SeNPs on the expression of MMPs may also be helpful in other carcinomas, as overexpression of MMP2 has been similarly found in bladder cancer [135], oral carcinoma [136], CRC [137,138], lung cancer [139], prostate cancer [140], as well as gastric cancer [141]. Se nanoparticles (80–220 nm) generated with the same species (*Bacillus* sp. MSh-1) were found to be almost 6-fold less cytotoxic to breast cancer cell MCF-7 compared to SeO_2_ at concentrations of 6.7 µg/mL. The same SeNPs also showed better DPPH scavenging activity and lower performance at 200 µg/mL, making them the expected choice for breast melanoma [31].

SeNPs produced by *Acinetobacter* sp. SW30 showed a selective cytotoxic effect on breast cancer cells (4T1, MCF-7) but not on non-cancer cells (NIH/3T3, HEK293). Therefore, they were recommended as a good choice for breast cancer cell selection. Oral administration (2.5 × 10^8^ CFU/mL) of *Lactobacillus plantarum* (ATCC 8014)-enriched SeNPs to mice demonstrated an effective immune response by promoting pro-inflammatory cytokines TNF-α, IFN-γ, and IL-2 in splenocytes and enhanced NK activity (natural killing) of cells [119,120]. Survival was significantly improved and tumor volume decreased compared to 4T1 breast cancer-bearing control mice [54]. Similarly, the purified SeNPs produced by the same probiotic bacteria were orally administered to 4T1 breast cancer-bearing mice to study the immune response [55]. In this study, the production of Th1 cytokines such as IL-12 and IFN-γ in spleen cells was enhanced in the test mice administered SeNPs. In addition, delayed hypersensitivity reaction (DTH) and survival were also significantly increased compared to control.

Yazdi et al. [53] used *Lactobacillus brevis* to produce SeNP-enriched biomass. A total of 0.5 mL orally administered biomass induced an efficient immune response by increasing IL-17 and IFN-γ levels with a remarkable increase in DTH responses and NK cytotoxicity in tumor-induced BALB/c mice. In addition, they also increased the survival rate and decreased the metastasis of liver tumors. Therefore, SeNPs containing biomass have been suggested as suitable candidates for the upcoming prevention and immunotherapy of breast tumors.

Another study conducted by Yazdi et al. showed that injection of purified SeNPs (produced by the same species; *Lactobacillus brevis*) and 4T1 cell antigen vaccine improved immune responses by increasing serum IL-2, IL-12, and IFN-γ levels and reduced TGF-β [56]. In addition, the vaccine demonstrated a stronger DTH response, reduced tumor volume, and prolonged survival in the mouse breast cancer model [57].

Similarly, Yazdi et al. investigated the immune responses with purified SeNPs produced by *Lactobacillus brevis* after oral administration of 100 µg/day in BALB/c mice after inducing breast cancer [57]. It was shown that cellular immunomodulatory components such as granzyme B, IL-2, IL-12, and IFN-γ were significantly improved in the mice treated with both SeNPs and crude antigens of 4T1 cells compared with the other groups (*p* < 0.05). However, TGF-β levels decreased in certain mice [57].

In the same year, Faghfuri et al. also studied SeNPs (50–250 nm) derived from *Lactobacillus brevis* [58]. Their results also showed an increase in an immunomodulatory component such as IFN-γ and the IFN-γ/IL-4 ratio at all doses administered compared to control doses. In addition, a lower tumor volume and prolonged survival were observed at a higher dose (200 mg/day) of SeNPs.

### 5.3. Effective against Prostate Cancer

Although prostate cancer is usually a slowly developing carcinoma, it is the third leading cause of cancer deaths in men, even in developed countries [142]. Each form of Se has been attributed to an anticarcinogenic effect with different mechanisms of action, and most of them have been restrained in prostate cancer [143,144]. SeNPs (40–180 nm) prepared by Sonkusre and Cameotra [36] using *Bacillus licheniformis* have been investigated for their potential use against prostate cancer. The harvested SeNPs induced necrosis in LNCaP-FGC cells without affecting red blood cell integrity at a nominal concentration of 2 μg/mL. A real-time gene expression study showed overexpression of TNF (tumor necrotic factor) and interferon regulatory factor (IRF) and a decrease in androgen receptor (AR) and prostate-specific antigen (PSA) expression. Significantly lower toxicity was observed after oral administration of a ten-fold higher concentration (50 mg Se/kg) of the same SeNPs in C3H/HeJ mice compared to 5 mg Se/kg L-selenomethionine [121].

Nanoparticles (40–180 nm) produced by *Bacillus licheniformis* JS2 promoted late necrosis or apoptosis in PC3 at a concentration of 2 µg/mL SeNPs, but no marked necrosis or apoptosis was detected in hPBMCs (normal cells) at the same concentration [122]. Later, similar SeNPs were reproduced by Sonkusre [120], and the anticancer mechanism was further investigated [36]. The results showed that the comparable SeNPs promoted ROS-mediated necroptosis in PC-3 cells at a Se concentration of 2 μg/mL by cellular internalization. The qPCR study showed an increase in gene expression of necroptosis-related IRF1 and TNF. Improved expression of RIP1 protein was also detected at the translational level. In addition, cell viability was significantly improved in the presence of the necroptosis inhibitor necrostatin-1.

### 5.4. Effective against Lung Cancer

Lung cancer is considered one of the most recurrent malignancies worldwide [145]. Treatment outcomes are among the poorest of all cancers, and survival is only 10–20% in five years [146]. SeNPs (50–80 nm particle size) produced anaerobically by *Bacillus licheniformis* ATCC 10716 have been shown to have a chemopreventive effect on lung cancer. The study aimed to test SeNPs as a chemotherapeutic agent against lung cancer under the influence of iron (III) nitrilotriacetate in male Wistar rats. Pretreatment (0.2 mg Se/kg body weight) with SeNPs significantly restored glutathione levels, catalase, and superoxide dismutase (SOD) activities and improved oxidative damage parameters such as lipid peroxidation, nitric oxide, and inflammatory factors such as C-reactive protein and TNF-α levels with improvement in the histological analysis of lung tissue and retraction of hyperplasia cells [52].

Bao et al. synthesized SeNPs (100–500 nm) using *Bacillus oryziterrae* and investigated their anticancer activity [122]. The results showed considerable inhibition of lung cancer cell line H157 in a dose-dependent manner, supporting a potential application of SeNPs in lung cancer treatment.

### 5.5. Effective against Hepatic Carcinoma

Hepatocellular carcinoma (HCC) is a cause of numerous cancer-related deaths worldwide. It is considered the most common carcinoma of the liver, originating from hepatocytes and occurring in nearly 80% of liver cancer cases [147]. Biogenic SeNPs with a particle size of 50–80 nm produced anaerobically by the probiotic *Lactobacillus casei* ATCC 393 showed significant inhibition of tumor development in the human liver cell line HepG2 and ameliorated diquat-induced oxidative stress in IPEC-J2 cells [124]. The same method for producing Se nanoparticles was applied and additionally enriched with proteins and polysaccharides to further investigate their antioxidant and hepatoprotective effects. The results of the study showed that SeNPs produced by *L. casei* 393 promoted apoptosis of HepG2 cells via caspase signaling cascade and endocytosis of SeNPs. Moreover, at a concentration of <25 μg Se/mL, they showed no cytotoxic effect on NCM460 proliferation and growth. Similarly, oxidative damage induced by H_2_O_2_ or diquat was ameliorated in intestinal epithelial cells, malondialdehyde (MDA) level was decreased, and GPx (glutathione peroxidase) activity was increased in the culture medium. These results indicate that the capped SeNPs produced by the probiotic *L. casei* 393 have a substantial effect on HCC [125].

### 5.6. Effective against Colon Cancer

Colorectal cancer (CRC) is the third most common disease in men and the second most common cancer in women. More than 1 million new cases are diagnosed each year, and nearly 0.5 million people die from it annually [148]. Even in the United States, 106,180 new cases of colon cancer and 44,850 cases of rectal cancer were reported in the current year, while 52,580 deaths were due to colon and rectal cancer combined [149]. SeNPs (170 –550 nm) produced by the probiotic strain *Lactobacillus casei* ATCC 393 were shown to inhibit colon cancer cell growth in vitro and in vivo. The specific SeNPs showed antiproliferative activity against CT26 colon cancer in mice. They also tended to induce apoptosis with an increased level of ROS in colon cancer cells, HT29 [118]. The same research group also confirmed the colorectal anticancer activity of SeNPs of the same species. These biogenic SeNPs have pro-apoptotic activity and the ability to promote immunogenic cell death (ICD) of colon cancer cells. In this in vitro study, the biomarkers involved in ICD (translocation of calreticulin and ERp57, the release of HMGB1 and ATP) were revealed, and the secretion of pro-inflammatory cytokines from cells treated with SeNPs was detected [119]. In addition, a study by Xu et al. [124] showed that SeNPs (50–80 nm) synthesized from the same *Lactobacillus* strain had a protective effect on the colon by stimulating the growth/proliferation of normal human IPEC-J2 cells, colon epithelial cells (NCM460), and human acute monocytic leukemia cell (THP-1)-derived macrophagocytes.

### 5.7. Effective as Antioxidants

All mammalian selenoproteins contain Se in the form of the amino acid selenocysteine (Sec), which is encoded by the UGA triplet. There are two forms of tRNA[Ser]Sec, which are essential for the synthesis of all selenoproteins. The tRNA[Ser]Sec isoforms are both the site of Sec synthesis and the adaptor molecules, which recognize the appropriate UGA codons in selenoprotein mRNAs. Twenty-two known eukaryotic selenoproteins are organized into distinct selenoprotein groups on the basis of the location and functional properties of Sec. GSH-Px, selenoprotein P, type I iodothyronine 5′-deiodinase (DI-I), and thioredoxin reductase (TR) have been characterized in animals and humans. Approximately half of the characterized selenoproteins have been implicated in having antioxidant functions. Thus, increased risks of human diseases associated with Se deficiency may be attributable to increased oxidative stress. Moreover, this oxidative stress and the resulting oxidative damage are important contributors to the formation and progression of cancer. However, Se supplementation can increase the level of enzymatic proteins, prevent the accumulation of free radical species, and reduce cellular damage [150,151,152,153,154,155,156]. ROS and RNS (reactive nitrogen species) are free radicals that form naturally and play an important role in normal cell physiology. However, in higher concentrations, these radicals can be harmful and damage key cellular components, especially proteins, DNA, and cell membranes [157,158,159]. The damage caused by reactive free radicals, especially DNA damage, is the cause of cancer and other health disorders [160,161]. Se is usually referred to as a nutritional antioxidant, but this effect is mainly attributed to selenium-containing proteins and not to pure Se [162]. SeNPs have been shown to have better antioxidant activity than other chemical forms of Se and also reduce the possibility of its toxicity [40].

Exopolysaccharide-capped biogenic SeNPs accumulated directly by *Bacillus paralicheniformis* SR14 (294 nm average particle size) showed greater antioxidant effects in scavenging ABTS, DPPH, and superoxide free radicals, but not OH radicals. In vitro studies with IPEC-J2 also showed remarkable cytoprotection of the same SeNPs against H_2_O_2_-induced oxidative stress, as they suppressed ROS formation. These effects suggest major antioxidant and cytoprotective properties of SeNPs for normal cells [126].

In another study by Xu et al., SeNPs were suggested to be a promising Se supplement with antioxidant and anti-inflammatory properties [126]. In this study, SeNPs (38–152 nm particle size) were biosynthesized using the probiotic *Lactococcus lactis* NZ9000 in an economical and environmentally friendly manner. These SeNPs significantly improved MDA (malondialdehyde) concentration and decreased GPx and total SOD activity in IPEC-J2 exposed to H_2_O_2_. Similarly, they prevented the H_2_O_2_-induced decrease in transepithelial electrical resistance and increased the FITC-dextran flux of IPEC-J2. Moreover, SeNPs decreased the increase in ROS, decreased mitochondrial membrane potential and ATP, and supported intestinal epithelial permeability in H2O2-exposed IPEC-J2 cells. Moreover, pretreated SeNPs attenuated the cytotoxic effect of *E. coli* (ETEC) K88 on IPEC-J2 cells and preserved the integrity of the intestinal epithelial barrier by upregulating occludin and claudin-1 expression along with a reduction in inflammatory cytokines.

Qiao et al. investigated in vivo the antioxidant parameters of biogenic SeNPs (50–80 nm) produced by the probiotic Lactobacillus casei ATCC 393 on diquat-induced intestinal barrier disruption and nuclear mechanisms in C57BL/6 mice [59]. Their results showed that oral administration of SeNPs significantly suppressed the increase in serum levels of alanine aminotransferase (ALT), aspartate aminotransferase (AST), diamine oxidase (DAO), and D-lactic acid promoted by diquat and improved the overall activities of SOD, GPx, and thioredoxin reductase (TrxR) in the jejunum and serum. In addition, they improved the number of goblet cells, decreased the formation of ROS, maintained mitochondrial function, and improved the expression of claudin-1 and occludin in the jejunum compared with the induced oxidative stress group model. In addition, SeNPs also stimulated nuclear factor Nrf2 and increased NADPH dehydrogenase and heme oxygenase. These results suggest that the above-mentioned SeNPs produced by *L. casei* ATCC 393 can protect intestinal barrier functions by counteracting oxidative damage induced by Nrf2 signaling.

## 6. Conclusions

SeNPs are expected to be the only candidate to replace the existing organic and inorganic Se species in clinical and nutritional practice due to their higher bioavailability and stability. SeNPs have received much attention as potential payloads to restore malignant growth. SeNPs prepared from probiotic/nonpathogenic bacteria with a particle size of 79–500 nm be effective against breast, prostate, lung, colon, and liver cancers and have equivalent antioxidant potential. They showed anticarcinogenic effects in breast tumors, mainly by ameliorating some pro-inflammatory cytokines IL-17, IL-2, IL-12, IFN-γ, and TNF-α, in addition to improving DTH and NK responses, decreased tumor volume, and prolonged survival in breast cancer animal model. The ameliorative effect of SeNPs in prostate cancer was indicated by overexpression of necroptosis related to IRF1 and TNF, a decrease in expression of PSA and AR, and an increase in ROS-mediated necroptosis in PC-3 cells. The antioxidant potential of probiotic SeNPs may also be an additionally anticancer, as they enhance ROS, SODG, and GPx activity induced by diquat/H_2_O_2_ and also exhibit scavenging activity for free radicals such as superoxide, DPPH, and ABTS. The present review has adequately demonstrated the recent importance, and anticancer effects of SeNPs biosynthesized from probiotic/nonpathogenic organisms. We hope that this information will support the safe and effective therapeutic use of SeNPs.

## Figures and Tables

**Table 1:** Summary of current work on SeNPs produced by probiotic/nonpathogenic bacteria with potential anticancer effects.

Probiotic	SeNPs Synthesizing Conditions	SeNPs Size (nm)	Cancer/Normal Cell Line	Method/Model	Dose	Anticarcinogenic Action/Major Outcomes	Reference
*Bacillus* sp. MSh-1	Aerobically grown in nutrient broth medium (pH 7) at 30 °C for 36 h in the presence of 281 mg SeO_2_/L, at 150 rpm	80–220	MCF-7/-	MTT, DPPH, RPA	6.7 µg/mL 200 µg/mL	Shown a greater DPPH scavenging action but lower reducing power than SeO_2_ at 200 µg/mL. Presented poor cytotoxicity on MCF-7 cell line compared to SeO_2_	[32]
*Bacillus* sp. MSh-1	Aerobically in nutrient broth (pH 7) at 30 °Cfor 36 h in the presence of 281 mg SeO_2_/L, at 150 rpm	80–220	HT-1080/-	MTT	10–100 μg/mL	Dose-dependent cytotoxicity. Inhibitory influence on the MMP-2 expression in the human HT-1080	[33]
*Bacillus licheniformis* JS2	Aerobically under the stress of 1.8 mM Na_2_SeO_3_, grown for 15 h at 200 rpm at 37 °C in TSB medium	40–180	PC-3/-		2 µg Se/mL	Induced ROS-mediated necroptosis. Increased expression of necroptosis-related tumor TNF and IRF1. Improved expression of RIP1 protein	[36]
*Bacillus licheniformis* ATCC 10716	Aerobically grown in nutrient broth containing 1 mM SeO_2_ at 37 °C for 24 h at 200 rpm	50–80	-	Animal model (Rats)	0.2 mg Se/kg (orally)	Chemo preventive effect on lung cancer tempted by iron ions. Reduced oxidative stress and inflammation markers of TN F-α and CRP	[52]
* Lactobacillus brevis *	Anaerobically grown in MRS broth for 72 h at 37 °C in the stress of 2.54 mM SeO_2_ solution	NA	-	Animal model (Mice)	0.5 mL of SeNP-enriched biomass (orally)	Shown a great level of IFN-γ and IL-17 with an elevation in DTH responses and NK cytotoxicity. Prolonged lifetime and drop in the malignant cancer metastasis	[53]
*Lactobacillus plantarum* (ATCC 8014)	Anaerobically grown under the stress of 2.54 mM SeO_2_ at 37 °C for 72 h	<250	-	Animal model (mice)	SeNPs enriched biomass (Orally)	Encourage an effective immune response via raise in pro-inflammatory cytokines IL-2, TNF-α, and IFN-γ, levels and improved in NK cell activity. Decreased tumor volumes and enhanced survival rate	[54]
*Lactobacillusplantarum* strain (ATCC 8014)	Anaerobically grown for 72 h in MRS broth at 37 °C in the existence of 200 mg/L SeO_2_	<250	-	Animal model (Mice)	100 μg/day (orally)	Increased creation of Th1 cytokines similar to IL-12 and IFN-γ in cells of spleen. Increased DTH response. Showed higher survival rate in animal breast cancer model	[55]
*Lactobacillus brevis*	Anaerobically grown at 37 °C for 72 h in 200 mg/L containing SeO_2_ MRS broth	NA	NA	Animal model (Mouse)	100 µg/mouse (inject)	Increased the amount of serum IL-2, IL-12 and IFN-γ and declined TGF-β in mice injected with SeNPs/vaccine. Lowered the tumor volume, improve DTH responses with a longer survival rate	[56]
* Lactobacillus brevis *	Anaerobically grown for 72 h in MRS broth at 37 °C, subsequently adding 200 mg/L of SeO_2_	-	-	Animal model (Mice)	100 µg/day (orally)	Increased significantly the levels of cellular immunomodulatory constituents such as IL-2, IL-12, granzyme B, and IFN-γ, whereas lowered the levels of TGF-β.	[57]
*Lactobacillus brevis*	Anaerobically grown for 72 h in MRS broth at 37 °C subsequently adding 200 mg/L of SeO_2_	50–250	-	Animal model (Mice)	200 mg/day (inject)	Increased IFN-γ and IFN-γ/IL-4 ratio. Lower tumor volume and prolonged survival	[58]
*Lactobacillus casei* ATCC 393	Anaerobically grown in MRS medium under the stress of 1.2 mM Na_2_SeO_3_ at 37 °C for 24 h	50–80	-	Animal model (Mice)	-	Inhibited the rise of ALT, AST, DAO and D-lactic acid amounts and improved T-SOD, TrxR and GPx activities. Improved the goblet cells number, decreased ROS, continued function of mitochondria. Enhanced the expression of claudin-1and occludin. Stimulated Nrf2 and enhanced NADPH dehydrogenase and heme oxygenase levels	[59]
*Lactobacillus casei* ATCC 393	Grown for 96 h in MRS broth without agitation at 37 °C under the stress of 20 mg/mL Se (NaHSeO_3_)	170–550	CT26, HT29	Animal model (male BALB/c mice)	6.5 mg/Kg	Exerted antiproliferative activity in CT26 murine colon cancer. Induced apoptosis and elevated reactive oxygen species levels in colon (HT29 cells) cancer cells	[118]
*Lactobacillus casei* ATCC 393	-do-	170–550	CT26, HT29, Caco-2	-	15 µg/mL	Inhibited the Caco-2 growth and induced activation of caspases 3/7/9. Induced apoptotic mechanisms in CT26 and HT29. Biomarkers, involving in ICD were detected	[119]
*Acinetobacter* sp. SW30	Grown aerobically in LB broth for 24 h at 30 °C and 180 rpm for 24 h under the stress of 1 mM Na_2_SeO_3_	79	4T1, MCF-7/NIH/3T3, HEK293	MTT	5–100 µg/mL	Selectively cytotoxic for 4T1, MCF-7 (breast cancer cells) but not for NIH/3T3, HEK293 (noncancerous cells)	[120]
*Bacillus licheniformis*	Aerobically grown in TSB under 1.8 mM Na_2_SeO_3_ stress. For 15 h at 200 rpm and 37 °C	40–180	LNCaP-FGC/-	XTT	2 µg Se/mL	Induced prostate cancer cell death via a TNF/IRF1-mediated necroptosis pathway and AR down-regulation	[121]
*Bacilluslicheniformis* JS2	Aerobically grown in TSB medium at 37 °C for 15 h under 1.8 mM Na_2_SeO_3_ stress, at 200 rpm	40–180	PC3/hPBMC	XTT	1–6 ug/mL	Inhibited propagation and prompting necrosis of human PC3 without producing somewhat major toxicity to noncancerous hPBMCs	[122]
*Bacillus* *oryziterrae* sp.	Cultivated anaerobically at 30 °C with selenite (1.0 mmol) in the dark.	100–500	H157/-	MTT	0.3 μg/μL	Dose-dependent inhibition of the lung cancer cells. Only <40% of cells survived exposed to 0.3 μg/μL wet of weight SeNPs	[123]
*Lactobacillus casei ATCC 393*	Grown in MRS containing 200 mg/mL of Na_2_SeO_3_ at 37 °C for 24 h without Shaking	50–80	HepG2/IPEC-J2, THP-1, NCM460	CCK-8 kit	-	Encouraged the growth and proliferation of IPEC-J2, NCM460, and THP-1. Repressed the growth of human HepG2, and improved diquat-induced oxidative upset in IPEC-J2	[124]
*Lactobacillus casei* 393	Anaerobically grown in MRS medium under the stress of 1.2 mM Na_2_SeO_3_ at 37 °C for 24	50–80	HepG2/NCM460	CCK-8 kit	0–100 µg/mL	Prompted HepG2 apoptosis. Alleviated diquat or H_2_O_2_ triggered oxidative destruction in NCM460 and condensed MDA concentration and improved GPx activity	[125]
*Bacillus paralicheniformis* SR14	Grown at 250 rpm and 37 °C for 72 h in medium including glucose, 2.0%; tryptone, 1.0%; yeast extract, 1.0%, K_2_HPO_4_, 0.1%; NaCl, 0.5%, MgSO_4,_ 1.5% including 5 mM Na_2_SeO_3_	294	/IPEC-J2 cells	MTT, ABTS, DPPH	-	Shown greater superoxide, DPPH, and ABTS free radicals scavenging activity, but not for OH radicals. Significant cytoprotective effect against H_2_O_2-_induced oxidative stress	[126]
*Lactococcus lactis* NZ9000	Grown in M17 broth comprising 0.5% glucose at 30 °C without any shaking under the stress of 0.6 mM of Na_2_SeO_3_ for 48 h	38–152	IPEC-J2/-	Antioxidant (In vivo)	0–64 µg/mL	Rise of MDA, the decrease in GPx and SOD activity. Prohibited the reduction of transepithelial electrical resistance. Revive FITC-dextran fluxes. Lessened ROS, reduction in membrane potential of mitochondria and ATP level and conserved intestinal epithelial permeability	[127]

NA: not available.
